# Teaching limited compression ultrasound to general practitioners reduces referrals of suspected DVT to a hospital: a retrospective cross-sectional study

**DOI:** 10.1186/s13089-021-00204-y

**Published:** 2021-02-02

**Authors:** Ossi Hannula, Ritva Vanninen, Suvi Rautiainen, Kalle Mattila, Harri Hyppölä

**Affiliations:** 1 Central Finland Central Hospital, Jyväskylä, Finland; 2 University of Eastern Finland, Kuopio, Finland; 3 Department of Clinical Radiology, Kuopio University Hospital, Kuopio, Finland; 4 Institute of Clinical Medicine, School of Medicine, University of Eastern Finland, Kuopio, Finland; 5 Pihlajalinna Medical Centre Eastern, Kuopio, Finland; 6 Emergency Department, Turku University Hospital, Turku, Finland; 7 University of Turku, Turku, Finland; 8 Emergency Department, South Savo Central Hospital, Mikkeli, Finland

**Keywords:** DVT, General practitioner, LCUS, POCUS, Postgraduate medical education, Primary health care

## Abstract

**Background:**

The aim of this study was to retrospectively determine whether teaching limited compression ultrasound (LCUS) to general practitioners (GP) would reduce the number of patients with a suspected lower extremity DVT referred to a hospital for ultrasound (US) examination. According to the current literature, an LCUS protocol is a safe way to diagnose or exclude lower extremity deep venous thrombosis (DVT) and a good option to radiologist-performed whole-leg ultrasound (US), especially in remote health care units where there may be a limited availability of radiological services.

**Methods:**

Between 2015 and 2016, altogether 13 GPs working in the same primary care unit were trained in LCUS for DVT diagnostics. The number of annual referrals due to a suspected DVT from Saarikka primary care unit to the closest hospital was evaluated before and after training. The incidence of DVT was considered to be constant. Thus, the reduction of referrals was attributed to the fact that these patients were diagnosed and treated in primary health care. Incidence rate ratio of hospital referrals was calculated. As a measure of safety, all patients diagnosed with a pulmonary embolism in the nearest hospital were evaluated to determine if they had undergone LCUS by a GP in primary care.

**Results:**

Before training in 2014, there were 60 annual referrals due to a suspected DVT; in 2017, after training, the number was reduced to 16, i.e., a 73.3% decrease. The incidence of referrals decreased from 3.21 to 0.89 per 1000 person-years. (IRR 3.58, 95% CI 2.04–6.66, *p* < 0.001). No patient with a pulmonary embolism diagnosis had LCUS performed previously, indicating that there were no false negatives, resulting in pulmonary embolism.

**Conclusions:**

Teaching LCUS to GPs can safely reduce the number of patients with a suspected DVT referred to a hospital substantially.

## Background

Lower extremity deep venous thrombosis (DVT) is a common disease with an estimated annual incidence of 1.2–1.6 per 1000 inhabitants [1, 2]. It is considered to be a continuum of the same disease as pulmonary embolism (PE) but with a different manifestation. Acute DVT often causes pain and swelling, and impairs the function of the leg and is frequently associated with a low fever. The most serious complication of DVT is its progression to PE with an incidence as high as 66% with the risk being higher in DVTs located in iliac or femoral veins as compared to those in the popliteal vein [3]. A significant proportion, 20–50%, of patients with a DVT develop post-thrombotic syndrome [4] often leading to a lower quality of life [5]. It is, therefore, vital to diagnose DVT accurately.

It is difficult to make a clinical diagnosis of DVT. The standard method for confirming or excluding DVT is Doppler compression ultrasound (US) examination, while venography is nowadays rarely performed. The accuracy of compression US in diagnosing a proximal DVT is good with a sensitivity of 93.8% and specificity of 97.8%, whereas in diagnosing a distal DVT (below the popliteal vein), the sensitivity decreases to 56.8%. Incorporating color Doppler increases sensitivity to 96.5% in proximal veins and to 71.2% in distal veins although with a lower specificity of 94.0% [6]. After a negative whole-leg compression US, only 0.5% of patients develop a thromboembolic complication during the following 3 months [7].

There is often a limited availability of radiological services in remote health care units. Most consultations to the hospital due to a suspected DVT require US examinations. These referrals to hospital are responsible for a significant use of health care resources, travel expenses, and loss of working time, and are time-consuming. Combining the clinical risk assessment ([modified] wells criteria) [8] with serum d-dimer testing can reduce the number of US examinations by 23% compared to only a clinical risk assessment [9]. Using an age-adjusted cut-off point for d-dimer can safely further reduce the number of US examinations by 15% [10].

Point-of-care ultrasound (POCUS) can be used as an alternative method to the standard venous US conducted by a radiologist. In a limited compression, US examination (LCUS, 2-point compression ultrasound, 2CUS) only common femoral, proximal superficial femoral, and popliteal veins are examined. LCUS is considered positive for thrombus if either the vein is not fully compressible or a thrombus is visualized [11–13]. LCUS is most often combined with a clinical pretest ([modified] wells criteria and d-dimer) [14]. LCUS has been shown to be a valuable tool in the diagnostic protocol of DVT [15–17]. LCUS performed by a heterogeneous group of emergency physicians had a sensitivity of 86% with a specificity of 93% as compared to a whole-leg compression US performed by a radiologist [18]. When a negative LCUS is repeated after approximately 1 week with another negative result, it has been shown safe to withhold anticoagulation treatment, i.e., there was only a 0.6% incidence of pulmonary embolism during the 3-month follow-up [19] paralleling the results achieved by whole-leg compression US [7].

Mumoli et al. performed a prospective cohort study in Italy during the years 2014–2016; this seems to be the only prospective controlled study on the accuracy of GPs performing LCUS. In their study, 18 GPs in ambulatory clinics with a rather extensive training evaluated a total of 1107 patients with symptoms indicative of a possible DVT. They performed an LCUS and regardless of the results referred them to a vascular clinic, where eight vascular US specialists performed a similar scan in a blinded manner. US examinations with negative or unclear results were repeated by the same operator after 5–7 days independently of the other group. In their study, GPs exhibited a diagnostic accuracy of 95.8%, with a sensitivity of 90.0%, and a specificity of 97.1% compared to vascular US experts [20].

Previous studies on LCUS have mainly been conducted in emergency departments in hospital settings. Their focus has been the accuracy of LCUS compared mainly to radiologist-performed US [18] and the effect of length of stay in the hospital emergency department [21]. In primary health care, it seems that the interest in LCUS and other types of point-of-care ultrasound examinations is growing [22]. There is only one previous study where GPs performed LCUS in primary health care [20]. As far as we are aware, there are no reports on the effect of training LCUS on the number of referrals.

The present study aimed to evaluate whether teaching GPs to use LCUS in primary care could reduce the number of suspected lower extremity DVT referrals to the secondary health care unit.

## Materials and methods

### Setting

Saarikka Primary Care Public Utility (a catchment area 18.000) is administered by five small communities in Central Finland. The primary health care center is in Saarijärvi with smaller centers in Kannonkoski, Karstula, Kivijärvi, and Kyyjärvi. The distance from Saarijärvi to the nearest secondary care hospital (Central Finland Central hospital, CFCH, with catchment area of 250,000) is 65 km, but it stretches up to 128 km from Kivijärvi. A total of 18 GPs are working in Saarikka. Three of them were skilled in LCUS before this study. A radiologist visits Saarijärvi weekly to conduct US examinations. Between these visits, there is no radiologist service available unless the patient is referred to the hospital. There are no other public or private health care facilities in the Saarikka area. When a DVT or PE is suspected, CFCH is the only hospital to which patients are referred.

### Study design and patients

The GPs working in Saarikka were trained using LCUS during 2015–2016 (see chapter “LCUS training”). In this study, we analyzed the number of patients referred to the hospital due to a suspected DVT before and after this training period.

The expected number of referrals was 104 annually. This number was reached by obtaining the number of radiologist-performed venous ultrasound exams in the hospital (1440 and 1445 in years 2015 and 2016, respectively) and comparing it to the population of Saarikka versus the hospital district (18.000–250.000, respectively). Since, according to our experience, almost every referral due to a suspected DVT leads to an US exam, and there are no other hospitals in this region, this number was considered to reflect the number of referrals to hospital. As the incidence of DVT was thought to be constant, it was hypothesized that any possible reduction in the number of referrals would be related to the training of GPs and their performance of LCUS examinations in Saarikka.

All referrals from Saarikka to CFCH during the reference period (2014) and post-intervention period (2017) were extracted from Saarikka patient information system. Urgent referrals were selected for further evaluation and reviewed in detail to determine the actual reason for referral. The referrals due to a suspected DVT were included in analysis. The referrals due to a suspected PE with or without DVT were excluded from the analysis, since the current practice demands that patients with a suspected PE are referred to hospital. There were no additional exclusion criteria.

As a secondary goal to assess the safety of LCUS, all patients diagnosed with a PE in CFCH during the study periods with an additional 3-month follow-up, 1.1.2014–31.3.2015 and 1.1.2017–31.3.2018, were screened from the CFCH records by selecting ICD-10 codes I26.0 and I26.9, indicating pulmonary embolism with or without acute cor pulmonale, respectively. Of these patients, those with a home address in Saarikka region were selected for further analysis and their patient records from Saarikka patient information system were reviewed for any mention of LCUS performed on them.

Since the radiologist still visits Saarikka weekly, all radiologists performed venous ultrasound in Saarikka were solicited from the PACS (picture archiving and communication system). These patients’ medical records were evaluated to see if there was an LCUS performed on them before the radiologist US and to compare the findings.

### LCUS training

During the years 2015–2016, a total of 18 physicians, medical students, or other short-term or part-time locums were trained to perform a LCUS examination by a physician with 5 years of experience in the diagnostic US as well as 7 years in emergency medicine (O.H.). The systematic LCUS training involved a 2-h training session including machine operating skills and fundamental US physics. Training included a lecture, demonstration, and a hands-on training on healthy volunteers. Pathological findings of thrombotic veins were shown in videos and as images. During the year 2016, the physicians in training were able to follow US examinations performed by the trainer and also to verify their clinical US findings with him. Of those physicians trained, eight GPs and five short-term locums continued to work in Saarikka during the post-intervention period. A total of four GPs did not participate the training. Additionally, five medical students working as GPs during summer 2017 were trained when they started working.

### LCUS protocol for suspected DVT

**Fig. 1:**
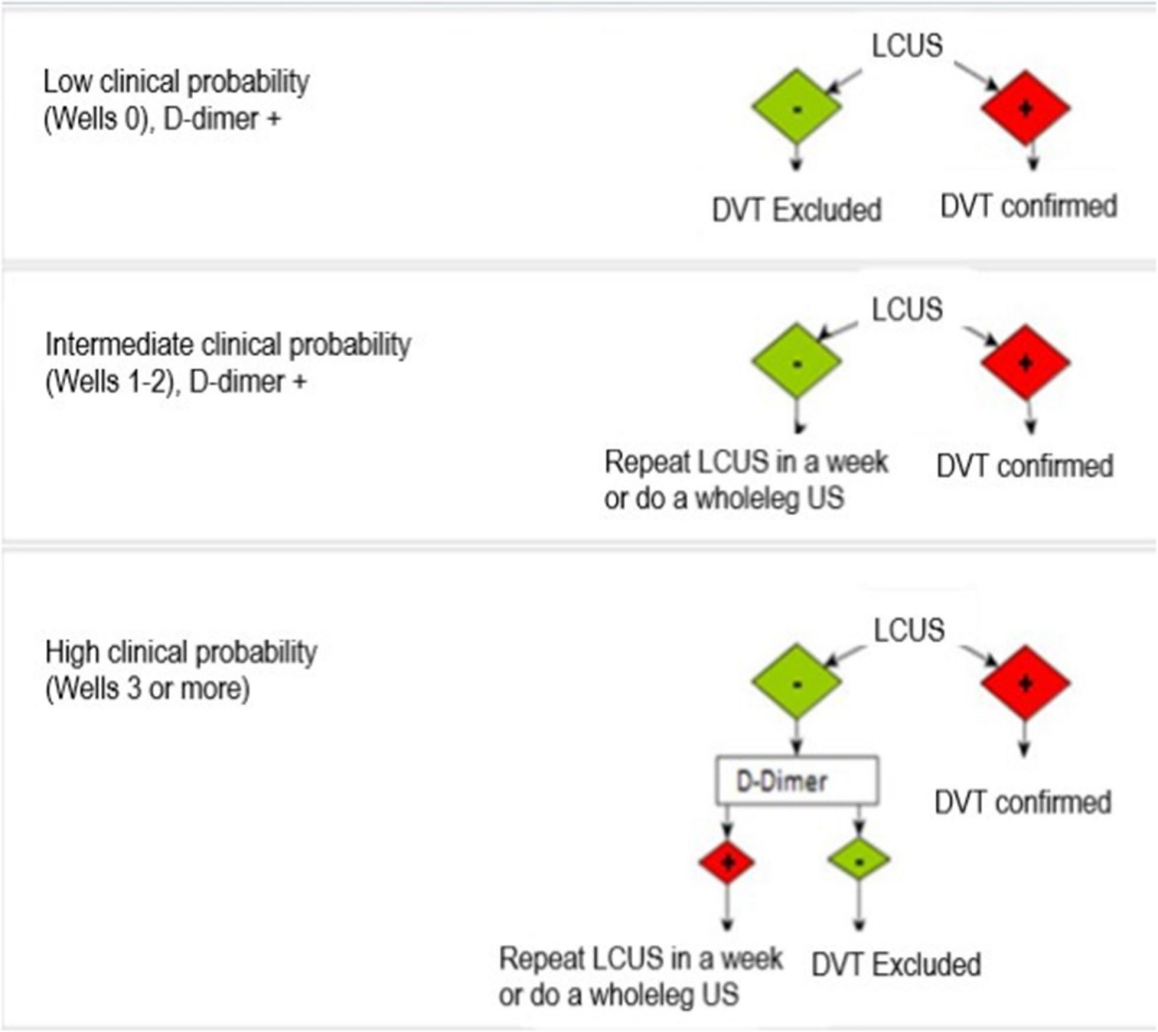
Diagnostic model for the use of LCUS in suspected DVT. Modified from Central Finland Central Hospital recommendation in accordance with national Current Care Guidelines

After the LCUS training period, the GPs were instructed to follow national guidelines [23]. This included a clinical risk assessment according to wells’ criteria [8], serum d-dimer testing with age-adjusted cut-off point [10], and LCUS. The protocol is illustrated in Fig. 1. In case the diagnostic image quality in LCUS was not considered sufficient to allow an accurate interpretation or if the performing GP was uncertain of the LCUS findings, the patient was referred to the hospital. Anticoagulation treatment was administered only in the case that a DVT was confirmed in a subsequent US exam.

### Statistics

The incidence rate ratio of referrals due to a suspected DVT was calculated with 95% confidence intervals with Stata 13.0 (StataCorp, Colleges Station, Texas 77845, USA). Statistical Product and Service Solutions (SPSS) 23.0 (Beijing Sichuang Weida Information Technology Co., Ltd., Beijing, China) was used for calculating the Pearson Chi-squared test for demographics data. A *p* value < 0.05 was considered a statistically significant difference.

## Results

During the reference period (2014), a total of 1313 urgent referrals were made from Saarikka to the Central Finland Central Hospital (Table 1) and 60 of those referrals were made due to a suspected DVT. During the post-interventional period (2017), there were a total of 1336 urgent referrals, of which 16 were made due to a suspected DVT, representing a decrease of 73.3% in annual references. The incidence of referrals per 1000 person-years decreased from 3.21 to 0.89. (IRR 3.58, 95% CI 2.04–6.66, *p* < 0.001).

**Table 1:** Demographics, number of urgent referrals, and referrals due to a suspected DVT from Saarikka to Central Finland Central Hospital

	2014	2017	*p*
Inhabitants	18,718	17,882	
Urgent referrals to included specialities	1313	1336	0.700
Suspected DVT referred to hospital	60	16	< 0.001
Age mean (SD)	67 (15)	72 (17)	0.226
Female	70%	69%	0.923

The annual number of PEs increased from 12 to 23. There were no LCUS performed on any of these patients.

The number of radiologist-performed US increased from 13 to 16. In 2014, two patients had an LCUS and radiologist-performed US, both with similar negative results. In 2017 two patients had both LCUS and radiologist perfomed US. One patient had negative results in LCUS, and in radiologist-performed US, the other had a negative LCUS in the common femoral vein region, but was inconclusive in the popliteal region and was hence referred to the radiologist, who found the common femoral vein region negative, but found a DVT in the low femoral and popliteal region.

The population decreased during the study years. However, compared to the population, the number of urgent referrals remained constant.

## Discussion

The key finding in this study was that teaching the LCUS protocol to GPs substantially reduced referrals to the hospital due to a suspected DVT. Importantly, it did not seem to result in an increasing number of pulmonary embolisms. This result was achieved with a relatively short training. In this study, we found that no DVTs were missed in patients with a diagnosed PE.

Some previous studies have implied that a DVT can mostly be diagnosed or ruled out using LCUS without the need for referral to a radiologist [15–17]. Though the accuracy of LCUS performed by a GP is lower than a whole-leg US performed by a radiologist [20], it does not seem to introduce additional complications presenting as PEs [19]. The diagnostic process can be undertaken in primary health care without referring the patients to the hospital which liberates hospital resources to other patients and thus saves a significant amount of time as well as costs. In addition, it probably increases patient satisfaction.

Since US is not a routine procedure performed every day by GPs, there is a risk that the adequate level of performance will not be maintained. Hence, it is essential to keep using US regularly. Although LCUS is not the only reason why GPs perform POCUS, the annual number of these other examinations is probably relatively low. It would help to preserve the quality if regular feedback from a consulting radiologist would be available and occasionally performing LCUS together with a peer GP would also be beneficial. A GP’s skill in performing and interpreting echocardiography findings is known to diminish significantly in 1–2 years if not practiced [24]. However, since LCUS has fewer objects that need to be interpreted, and the diagnostic image is easier to acquire than in echocardiography, results related to echocardiography may not be fully generalizable to LCUS skills. There are no studies on the accuracy of LCUS years after training.

It seems that GPs can achieve a high diagnostic accuracy in LCUS [20]. Our study highlights that even after the actual study period, GPs still refer fewer patients to radiologists than they did before the teaching intervention.

The strength of this study is its stable study environment. During the study period, there have been no significant changes in primary care in Saarikka. Even though there were already a few GPs performing LCUS before the study, the number of DVT referrals was large enough for the intervention to make a statistical difference.

Our study has several limitations. The retrospective study period for this relatively small population is rather short, and the numbers of referrals before and after the intervention were low. Nevertheless, even in this small study, the reduction in referrals proved to be statistically significant. Before this study, based on the region’s population, it could be predicted that a total of 104 patients would require an urgent US exam due to suspected DVT, but in the year 2014, the actual number was 60. This difference has two likely explanations; there were already three GPs performing LCUS during the reference period, one working full-time and two part-time. Though the number of LCUS performed by these GPs is unknown, they are likely to have reduced the referrals to hospital. Additionally, 13 patients were seen by the weekly visiting radiologist without referring them to the hospital. Considering these facts, the amount of patients with suspected DVT seems to be about the predicted number. As the amount of radiologist-performed US remained the same (*n* = 13–16), and the incidence of a suspected DVT can be assumed to remain constant in this population, it is reasonable to assume that the substantial reduction in the number of referrals was attributable to the increasing use of LCUS in DVT diagnostics.

The exact number of LCUS examinations performed is unknown, since there was no structured method to register how many procedures were performed. The number of possible false-positive examinations is also unknown, as the positive US findings were not verified by other examinations. However, all diagnosed PEs in CFCH for patients from Saarikka area were evaluated further, and no false-negative LCUS findings in these patients were detected. None of the patients referred to the hospital had undergone a previous LCUS exam.

It is possible that some patients could have been diagnosed with a DVT in health care facilities in other parts of Finland. It is virtually impossible to retrospectively pinpoint these patients. It was assumed that the number of these patients would remain constant and low in this stable population, thus most probably not interfering with the results of this study.

The reason for the increase in the number of PEs remains unclear. However, the PEs were not the goal of this study. Since there was no prior LCUS conducted on these patients, it is not considered to be related to the increase in LCUS examinations.

In future studies, it would be interesting to evaluate the long-term effect on the accuracy of LCUS years after a single training session. In addition, a study in a primary care facility where no LCUS is made before training would give more information on the true significance associated with this kind of training. Furthermore, a longer follow-up time with a larger population would provide more statistical power. A cost-minimization analysis could help further justify this diagnostic pathway.

No misdiagnoses or complications were found in this study. However, no conclusions on the safety aspects of LCUS performed by GPs can be made solely from this study, clarification of this issue will require future conformation. In previous studies, the sensitivity and specificity of LCUS performed by EPs or GPs have been found to be lower than those of a radiologist performing whole-leg US examination. Even though it seems that a repeated LCUS is as safe as a single whole-leg US, the data are rather scarce. The safety of LCUS should be evaluated in further studies by examining the amount of PEs after negative repeated LCUS and also the incidence of the post-thrombotic syndrome.

## Conclusion

This study shows that teaching GPs LCUS may safely and significantly reduce hospital referrals due to a suspected lower extremity DVT. It seems to be possible to achieve this benefit with a relatively low teaching input. In our study, we found no missed DVTs in patients with PE, in accordance with the previous data. Since most GPs perform a relatively low number of any type of ultrasound examinations, the long-term accuracy of LCUS years after training remains unclear and should be a focus of a controlled study in the future. A prospective study with a larger population, especially one in which the GPs have no previous experience with LCUS, would reveal the full effect of training on the number of referrals.

## Data Availability

The data that support the findings of this study are available from hospital records of Central Finland Central Hospital and Saarikka Primary Care Public Utility, but restrictions apply to the availability of these data, which were used under license for the current study, and so are not publicly available. Data are, however, available from the authors upon reasonable request and with permission of Northern Savo hospital district ethical board, chief physician of Central Finland Central Hospital, and chief physician of Saarikka Primary Care Public Utility.

## Abbreviations

GP: General practitioner
LCUS: Limited compression ultrasound
POCUS: Point-of-care ultrasound
EP: Emergency physician
PE: Pulmonary embolism
DVT: Deep venous thrombosis
US: Ultrasound
CFCH: Central Finland Central Hospital
PACS: Picture archiving and communication systems
